# Supplementary material for: The biochemical effects of carotenoids in orange carrots on the colonic proteome in a mouse model of diet-induced obesity
Source: Front Nutr. 2024 Nov 11;11:1492380. doi: 10.3389/fnut.2024.1492380 (PMC11587903; doi:10.3389/fnut.2024.1492380)
Supplement: Supplementary file 1 [file Data_Sheet_1.pdf]

Supplementary Materials

A) Diet Pellet Composition

	D12450HB (LFD)	D12451B (HFD)	White Carrot (HFD+WC)	Orange Carrot (HFD+OC)
**Ingredient**	**gm**	**gm**	**gm**	**Gm**
Casein	200	200	**186.7**	**186.7**
L-Cystine	3	3	3	3
Corn Starch	452.2	72.8	**0**	**0**
Maltodextrin 10	75	100	**72.7**	**72.7**
Sucrose	172.8	172.8	172.8	172.8
Cellulose, BW200	50	50	**9.1**	**9.1**
Soybean Oil	25	25	25	25
Lard	20	177.5	177.5	177.5
Mineral Mix, S10026	10	10	10	10
Dicalcium Phosphate	13	13	13	13
Calcium Carbonate	5.5	5.5	5.5	5.5
Potassium Citrate, 1 H2O	16.5	16.5	16.5	16.5
**Vitamin Mix, V10001**	10	10	10	10
Choline Bitartrate	2	2	2	2
FD&C Blue Dye #1	0.05	0.05	0.05	0.05
**White carrot powder**	**0**	**0**	**175.6**	**0**
**Orange carrot powder**	**0**	**0**	**0**	**175.6**
**Total**	**1055.05**	**858.15**	**879.45**	**879.45**
**Gm**				
Protein	177.0	177.0	177.0	177.0
Carbohydrate	710.0	355.6	355.6	355.6
Fat	45.0	202.5	202.5	202.5
Fiber	50.0	50.0	50.0	50.0
**gm%**				
Protein	16.8	20.6	20.1	20.1
Carbohydrate	67.3	41.4	40.4	40.4
Fat	4.3	23.6	23.0	23.0
**Carrot Powder**	**0.0**	**0.0**	**20.0**	**20.0**
**Kcal**				
Protein	708	708	708	708
Carbohydrate	2840	1422.4	1422	1422
Fat	405	1822.5	1823	1823
Total	3953	3953	3953	3953
**kcal%**				

Protein	**18**	**18**	**18**	**18**
Carbohydrate	**72**	**36**	**36**	**36**
Fat	**10**	**46**	**46**	**46**
**kcal/gm**	3.75	4.61	4.49	4.49

B) Vitamin Mix V10001

**Carbohydrate**	Sucrose, Fine Granulated	78.42 g
**Vitamin**	Vitamin E Acetate, 50%	10.00 g
**Vitamin**	Niacin (a.k.a. B3, Nicotinic Acid)	3.00 g
**Vitamin**	Biotin, 1%	2.00 g
**Vitamin**	Pantothenic Acid, d, Calcium (a.k.a. B5)	1.60 g
**Vitamin**	Vitamin D3, 100,000 IU/gm	1.00 g
**Vitamin**	Vitamin B12, Cyanocobalamin, 0.1%	1.00 g
**Vitamin**	Vitamin A Acetate, 500,000 IU/gm	0.80 g
**Vitamin**	Pyridoxine HCl (a.k.a. B6)	0.70 g
**Vitamin**	Riboflavin (A.K.A. B2)	0.60 g
**Vitamin**	Thiamine HCl (a.k.a. B1)	0.60 g
**Vitamin**	Folic Acid	0.20 g
**Vitamin**	Menadione Sodium Bisulfite	0.08 g
	Total:	100.00 g

Supplementary Table 1: Ingredients of experimental diet pellets. Compositional breakdown of **A)** dietary pellets and **B)** vitamin (V10001) mix formulated by Research Diets, Inc.

Supplementary Table 2

Fraction No.	ACN (%)	ACN (μL)	TEA (0.1%) (μL)
Wash	5.0	50	950
1	10.0	100	900
2	12.5	125	875
3	15.0	150	850
4	17.5	175	825
5	20.0	200	800
6	22.5	225	775
7	25.0	250	750
8	50.0	500	500

Supplementary Table 2: Fractionation of TMT-labeled peptides. Volume of LC-MS grade acetonitrile (ACN) and triethylamine (TEA) to fractionate TMT-labeled peptides. Fraction 8 was chosen for the proteomic LC-MS/MS analysis.

		-2 in %		-1 in %		M+ in %	+1 in %		+2 in %	
**Mass Tag**	**Reporter Ion Mass**	**-2x13C**	**-13C-15N**	**-13C**	**-15N**	**-**	**+15N**	**+13C**	**+15N+13C**	**+2x13C**
126	126.127726	0	0	0	0	100%	0.31	9.09	0.02	0.32
127N	127.124761	0	0	0	0.57	100%	0	9.79	0	0.33
127C	127.131081	0	0	0.84	0	100%	0.23	8.4	0.02	0.27
128N	128.128116	0	0	0.82	0.65	100%	0	8.13	0	0.26
128C	128.134436	0	0	1.44	0	100%	0.34	6.26	0	0.17
129N	129.131471	0	0.14	1.3	0.89	100%	0	7.52	0	0.12
129C	129.13779	0.13	0	2.59	0	100%	0.32	6.07	0.01	0.09
130N	130.134825	0.13	0	2.41	0.27	100%	0	5.58	0	0.1
130C	130.141145	0.25	0	3.22	0	100%	0.28	5.06	0	0.06
131N	131.13818	0.03	0	2.78	0.63	100%	0	4.57	0	0.12
131C	131.144499	0.08	0	3.94	0	100%	0.45	3.33	0	0.02
132N	132.141535	0.07	0.01	3.14	0.73	100%	0	3.4	0	0.03
132C	132.147855	0.08	0	3.65	0	100%	0.5	1.97	0	0
133N	133.14489	0.15	0.01	3.58	0.72	100%	0	1.8	0	0
133C	133.15121	0.18	0	4.14	0	100%	0.4	1.11	0	0
134N	134.148245	0.3	0.03	5.49	0.62	100%	0	1.14	0	0

Supplementary Table 3: The quantification method used in PD incorporated the parameters for reporter ions and isotope correction factors as defined by the product data sheet for TMTpro product number A44521, lot no. YG373065.

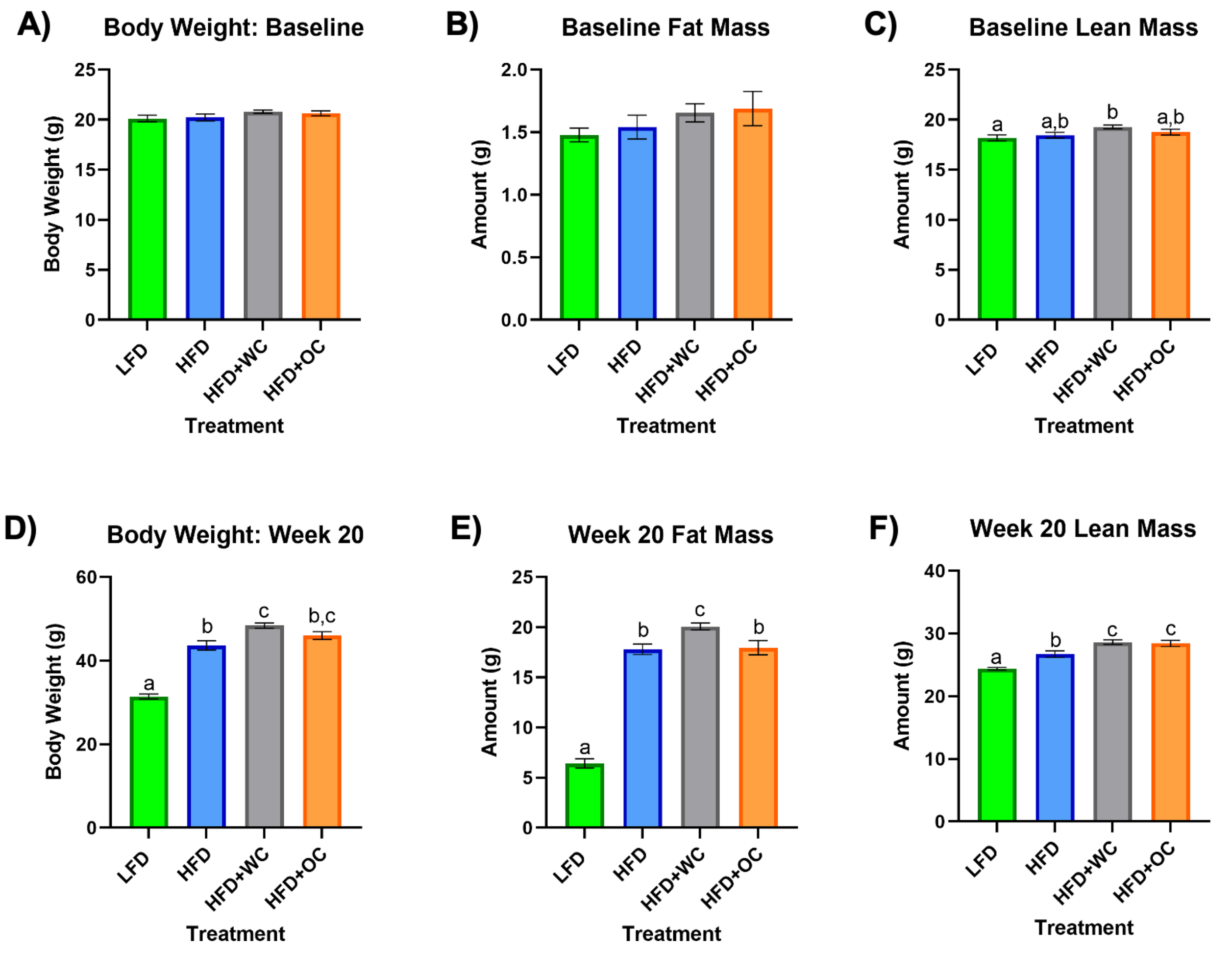

Supplementary Figure 1: Individual measurements of body composition at baseline and endpoint. **A-C)** Baseline measurements of **A)** body weight, **B)** fat mass, and **C)** lean mass at Week 0 before dietary intervention. **D-F)** Endpoint measurements of **D)** body weight, **E)** fat mass, and **F)** lean mass at Week 20. Fat mass (**B&E)** and lean mass (**C&F)** measurements were determined by EchoMRI. LFD, HFD, HFD+WC, HFD+OC n=20. Values are means ± SEM. Body weight, fat mass, and lean mass measurements were analyzed by one-way ANOVA with post-hoc Tukey HSD. Letter differences indicate statistical significance ($p < 0.05$).

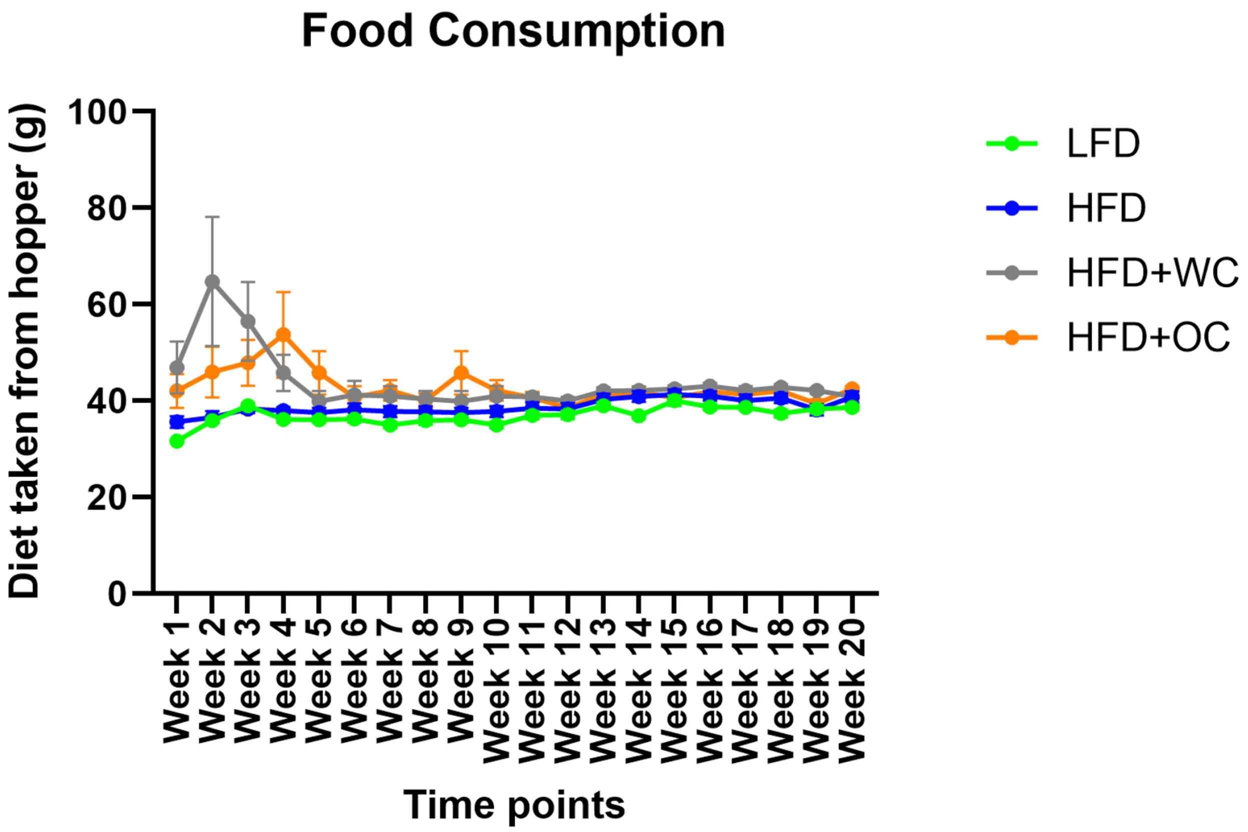

Supplementary Figure 2: Weekly food consumption. Measurements of diet pellets taken from the food hopper in the cages on a weekly basis. Weight of pellets in the hopper only (not including any food on the floor of the cage) was recorded at the time of administration and then the amount leftover when the week elapsed, reporting the difference between the two points. A two-way mixed ANOVA with post hoc Tukey HSD to illustrate the differences in food consumption over time.

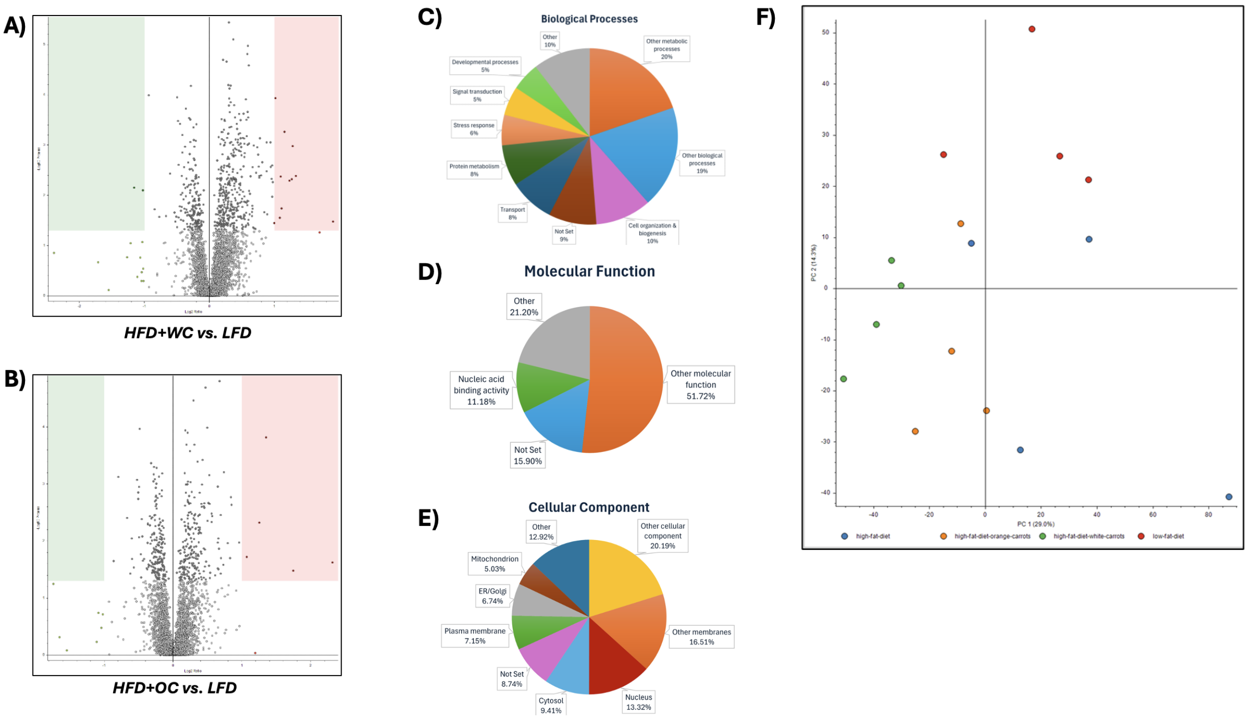

Supplementary Figure 3: Additional comparisons from quantitative proteomics. A-B) Volcano plots of differentially expressed peptides upon comparing abundances between **A)** HFD+WC vs. LFD and **B)** HFD+OC vs. LFD. **C-E)** pie charts of identified protein KEGG characteristics, **C)** Biological Process, **D)** Molecular function, **E)** Cellular Component. **F)** PCA plot of identified peptides across treatment groups

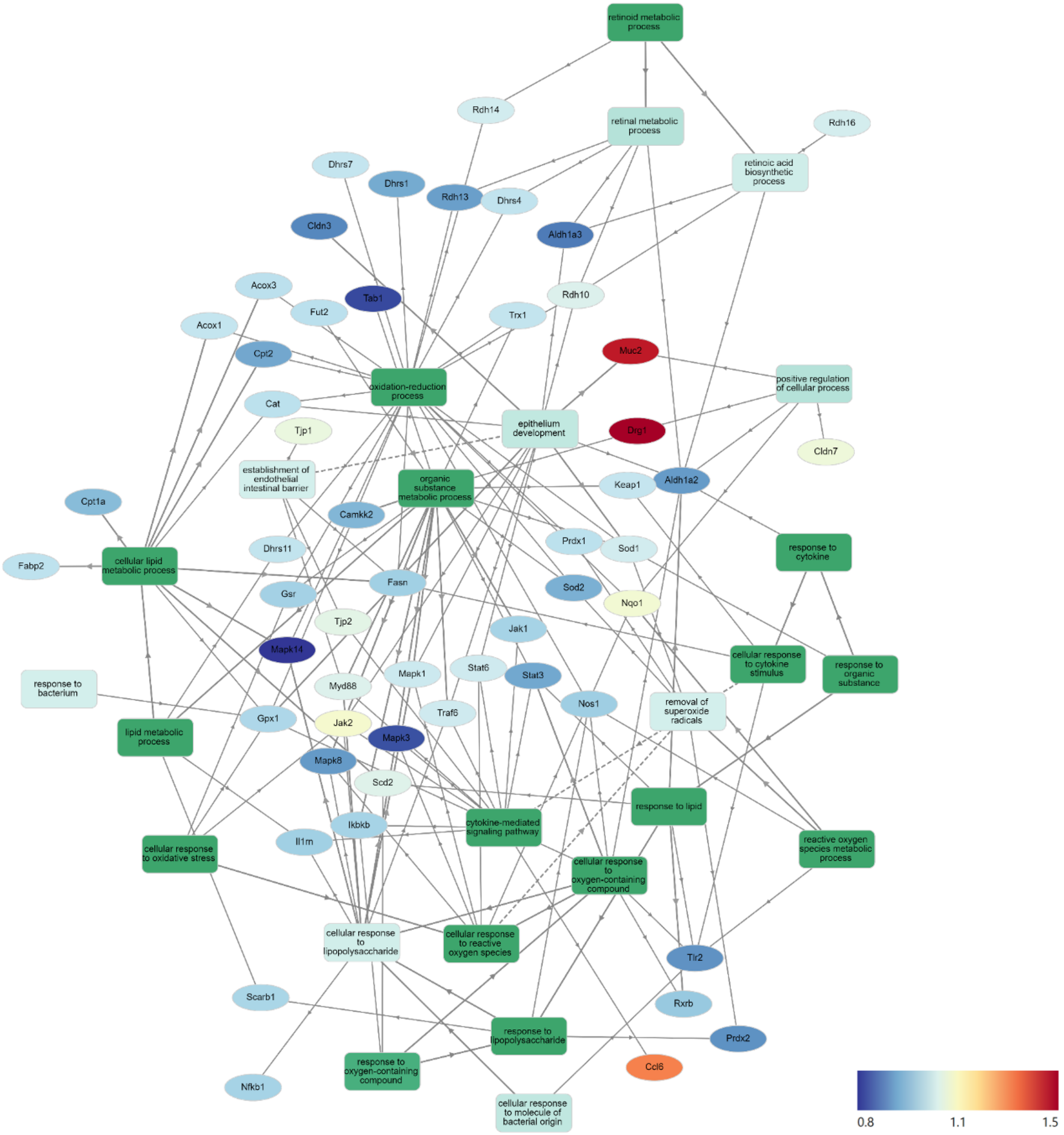

Supplementary Figure 4: GOnet Map of HFD+OC/HFD+WC comparison. Gene Ontology Network (GOnet) map of the same proteins depicted in Figure 4 with a relative quantitative comparison between HFD+OC versus HFD+WC (color of protein nodes and legend correspond to HFD+OC/HFD+WC fold change). KEGG Pathway: Biological Process
